# Mechanisms underlying neuro-inflammation and neurodevelopmental toxicity in the mouse neocortex following prenatal exposure to ethanol

**DOI:** 10.1038/s41598-017-04289-1

**Published:** 2017-07-10

**Authors:** Munekazu Komada, Nao Hara, Satoko Kawachi, Kota Kawachi, Nao Kagawa, Tetsuji Nagao, Yayoi Ikeda

**Affiliations:** 1 Department of Anatomy, School of Dentistry, Aichi Gakuin University, 1-100 Kusumoto-cho, Chikusa-ku, Nagoya, Aichi 464-8650 Japan; 2 Department of Life Science, Kindai University, 3-4-1 Kowakae, Higashiosaka, Osaka, 577-8502 Japan

## Abstract

Fetal alcohol spectrum disorders (FASD) constitute a wide range of disorders that arise from prenatal exposure to ethanol (EtOH). However, detailed reports regarding the adverse effects of prenatal EtOH exposure on neocortical morphology and its underlying pathogenic mechanisms are limited. In the present study, we aimed to characterize the anatomical abnormalities of neocortical development and their correlation with microglial properties and neuro-inflammation in a mouse model of FASD. We evaluated the development and maturation of the neocortex in ICR mice prenatally exposed to 25% (w/v) EtOH using histological and molecular analyses. Reduced proliferation and excessive cell death were observed in the dorsal telencephalon. Abnormal neuronal distribution, layer formation, and dopaminergic neuronal projections were observed in the neocortex. Disruption of microglial differentiation (M1/M2 microglial ratio) and abnormal expression of pro-inflammatory and neurotrophic factors were induced, and these abnormalities were ameliorated by co-treatment with an anti-inflammatory drug (pioglitazone). FASD model mice displayed histological abnormalities, microglial abnormalities, and neuro-inflammation in both the embryonic and newborn stages. Thus, anti-inflammatory therapeutics may provide a novel preventive approach for the treatment of FASD.

## Introduction

Fetal alcohol spectrum disorders (FASD) comprise a group of related disorders and conditions induced by prenatal exposure to ethanol (EtOH) resulting from maternal consumption. A vast array of adverse health effects—including mental, emotional, craniofacial, physiological, and immune disorders—have been documented, and research has revealed that EtOH induces apoptosis and inhibits neural stem cell proliferation in developing brain^1, 2^. Prenatal exposure to EtOH affects radial glial cell morphology, neuronal migration^3^, and induces cell death in layer V neurons^4^, particularly in the dorsal telencephalon. Recent research has also revealed that perinatal exposure to EtOH affects microglia during brain development^5^.

Microglia originate from immature erythromyeloid progenitors and perform phagocytic functions in the central nervous system (CNS)^6^. Microglia act as the principle immune cells in the brain, phagocytize apoptotic cells, mediate cell death signaling, and are important for proper neocortical development^6, 7^. In addition, microglia promote proliferation and differentiation of neural stem cells during CNS development^7, 8^. In the developing mouse neocortex, microglia are morphologically amoeboid and transition to a ramified morphology in the early postnatal period^8^.

Microglia are activated by a variety of inflammatory factors, each associated with a different pattern of response. M1 “pro-inflammatory” microglial activation plays a vital role in the defense against pathogens by producing pro-inflammatory cytokines such as interleukin 1 beta (IL1β), tumor necrosis factor alpha (TNFα), IL6, and IL12^9^. In contrast, M2 “anti-inflammatory” microglial activation promotes tissue remodeling/repair and releases anti-inflammatory cytokines such as IL10, IL4, and transforming growth factor beta (TGFβ)^9^. In addition, M2 microglia produce numerous neuronal protective and trophic factors, such as insulin-like growth factor 1 (IGF1) and brain-derived neurotrophic factor (BDNF)^9^. M1 and M2 microglia have different properties and are associated with different functions in response to pathological alterations in the injured brain. In addition, Cx3Cl1 (also known as fractalkine or chemokine ligand 1) signaling is associated with both neuroprotection (anti-inflammatory responses) and pro-inflammatory responses, following the activation of M1 or M2 microglia, respectively^10^.

The symptoms of FASD include developmental disorders and learning disabilities. Among the brain regions prominently affected by prenatal EtOH exposure is the neocortex, which coordinates higher cerebral functions. Analyses of the adverse effects of prenatal EtOH treatment on the developing neocortex are important for clarifying the pathogenic mechanisms of developmental disorders and intellectual impairment in FASD. However, detailed knowledge of the toxic effects of prenatal EtOH exposure on cellular and developmental phenotypes in the neocortex is lacking. In addition, EtOH exposure has been reported to induce neuro-inflammation as well as excessive activation and apoptosis of microglia in the developing rodent neocortex^11–14^. However, the specific abnormalities and underlying pathogenic molecular mechanisms associated with functional microglial differentiation (M1 versus M2) and morphology in mice subjected to prenatal EtOH exposure remain to be elucidated. Therefore, in the present study, we aimed to elucidate the pathogenic cellular and molecular mechanisms underlying the adverse effects associated with prenatal EtOH exposure and suggest a potential role for anti-inflammatory treatment in the prevention of FASD.

## Results

### Clinical signs/reproductive performance of pregnant mice exposed to EtOH

Pregnant females exhibited decreased motor activity and gait disturbances for 20–30 min after each administration of EtOH at 2 g∙kg^−1^ during the dosing period. All dams successfully delivered pups on embryonic day (E) 19, and no change was observed in the number of live newborns on postnatal day (P) 0 in any group. Furthermore, all dams nursed their pups normally until P21, and the viability of pups (after adjustment of pup number on P1) in the EtOH-exposed groups was comparable to that of the controls.

### Decreased body and brain weight of newborns prenatally exposed to EtOH

The body weight of P3 newborns exposed to EtOH in the prenatal stage (1 or 2 g∙kg^−1^∙day^−1^) exhibited a tendency for dose-dependent reductions (Table 1), and their absolute brain weight decreased more significantly than that of controls (Table 1). Therefore, the relative brain weights of EtOH-treated and control mice did not differ significantly (Table 1). Furthermore, the body weights of prenatal-EtOH-exposed male pups from P1 through 11 weeks of age were lower than those of controls (Table 2).

**Table 1:** Body and brain weights of P3 newborns prenatally exposed to ethanol (EtOH).

Group	Sex	Body weight (g, A)	Brain weight	
			Absolute (g, B)	Relative (%, B/A)
Control	Male	3.16 ± 0.180	0.174 ± 0.012	5.37 ± 0.266
EtOH 1 g∙kg^−1^	Male	3.07 ± 0.203	0.159 ± 0.008*	5.10 ± 0.542
EtOH 2 g∙kg^−1^	Male	3.05 ± 0.282	0.144 ± 0.008*	5.13 ± 0.270

^*^P < 0.05, n = 18.

**Table 2:** Body weight change of offspring prenatally exposed to ethanol (EtOH).

Group	P 1	5 weeks	7 weeks	11 weeks
Control	2.08 ± 0.16	37.02 ± 2.77	41.37 ± 3.51	46.06 ± 5.65
EtOH 1 g∙kg^−1^	2.07 ± 0.18	35.43 ± 3.43	38.10 ± 2.89*	42.62 ± 4.01*
EtOH 2 g∙kg^−1^	2.00 ± 0.15	34.42 ± 2.70*	36.05 ± 4.65*	39.70 ± 4.96*

*P < 0.05, n = 18.

### Effect of EtOH exposure on embryonic cell proliferation and survival

**Figure 1:**
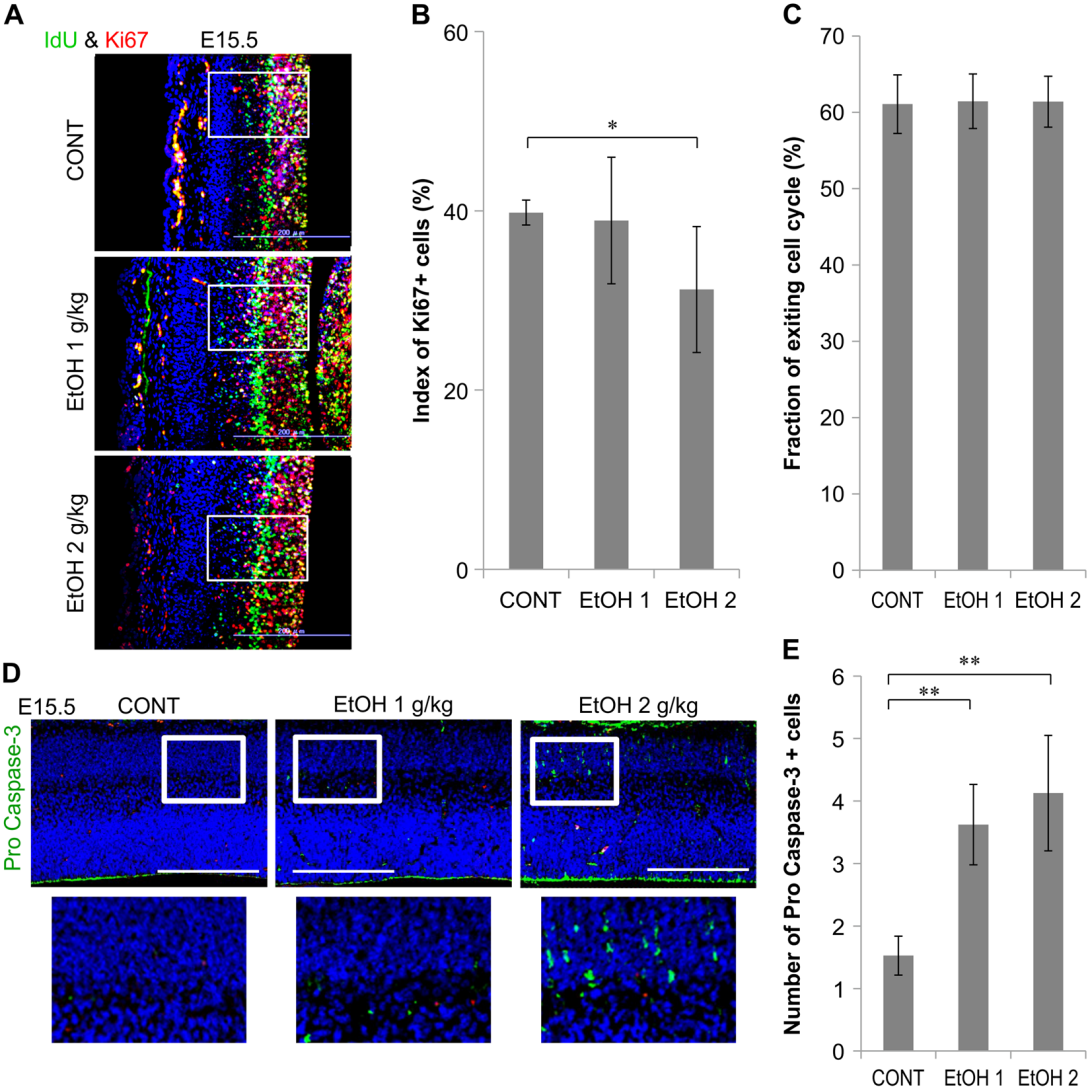
Decreased proliferation of neural stem cells and increased apoptosis in the embryonic dorsal telencephalon due to prenatal EtOH exposure. (**A**) Images showing parasagittal brain sections of E15.5 embryos stained with anti-Ki67 and anti-IdU antibodies 24 h after IdU pulse labeling in control (n = 6) and EtOH-treated embryos (n = 6, respectively). Images were used to estimate proliferation and cell cycle exit (neurogenesis). (**B**) Graph depicting the number of Ki67 + cells (red and yellow in A, white boxes in A) in E15.5 embryos. (**C**) Graph displaying the ratio of IdU + /Ki67− (green, a measure of the absence of cell division, white boxes in A) to all cells labeled with IdU (green and yellow) 24 h after labeling. This ratio was used as a measure of cell cycle exit. (**D**) Images of parasagittal sections from E15.5 (n = 6) and EtOH-treated embryos (n = 6, respectively) stained with anti-active & pro-caspase-3 antibody. White boxes indicate the regions from which high magnification images in the bottom row were taken. (**E**) Graph depicting the quantification of cells in the whole image. Scale bar, 200 µm. *P < 0.05, **P < 0.01, compared to 0 mg∙kg^−1^ controls using Dunnett’s test or Steel’s test.

We first examined neural stem cell proliferation via immunostaining with anti-Ki67 (a nucleoprotein expressed in proliferative cells) antibody (Fig. 1A; high magnification images in Sup. Fig. S1A, Supplemental Methods). The Ki67-positive (Ki67+) cell index (Ki67 + cells/DAPI + cells × 100) decreased more significantly in the dorsal telencephalon of 2 g∙kg^−1^ EtOH-treated embryos than in controls on E15.5 (Fig. 1B).

Secondly, we investigated neural stem cell neurogenesis by examining cell cycle exit via IdU-labeling analysis. We double-labeled cells using anti-Ki67 and anti-IdU (a thymidine analog) antibodies 24 h after IdU labeling. Cell cycle exit was determined as the ratio of cells that exited the cell cycle (IdU+ and Ki67, indicating no cell division) to all cells labeled with IdU 24 h after labeling (Fig. 1A; high magnification images in Sup. Fig. S1A). Quantification revealed no significant differences in cell cycle exit between EtOH-treated and control embryos on E15.5 (Fig. 1C).

We examined the induction of apoptosis in the dorsal telencephalon of E15.5 embryos exposed to EtOH (1 and 2 g∙kg∙^−1^day^−1^). Analysis of the relevant immunostaining results revealed significantly increased numbers of active & pro-caspase-3+ cells (Fig. 1D; high magnification images in Sup. Fig. S1B) in the developing dorsal telencephalon of embryos treated with 1 and 2 g∙kg^−1^ of EtOH relative to controls at E15.5 (Fig. 1E). These data suggest that the mouse models of FASD utilized in the present study exhibited abnormal cell proliferation and survival without changes in neurogenesis during neocortical development at E15.5.

### Abnormal neocortical neuron distribution in newborns prenatally exposed to EtOH

**Figure 2:**
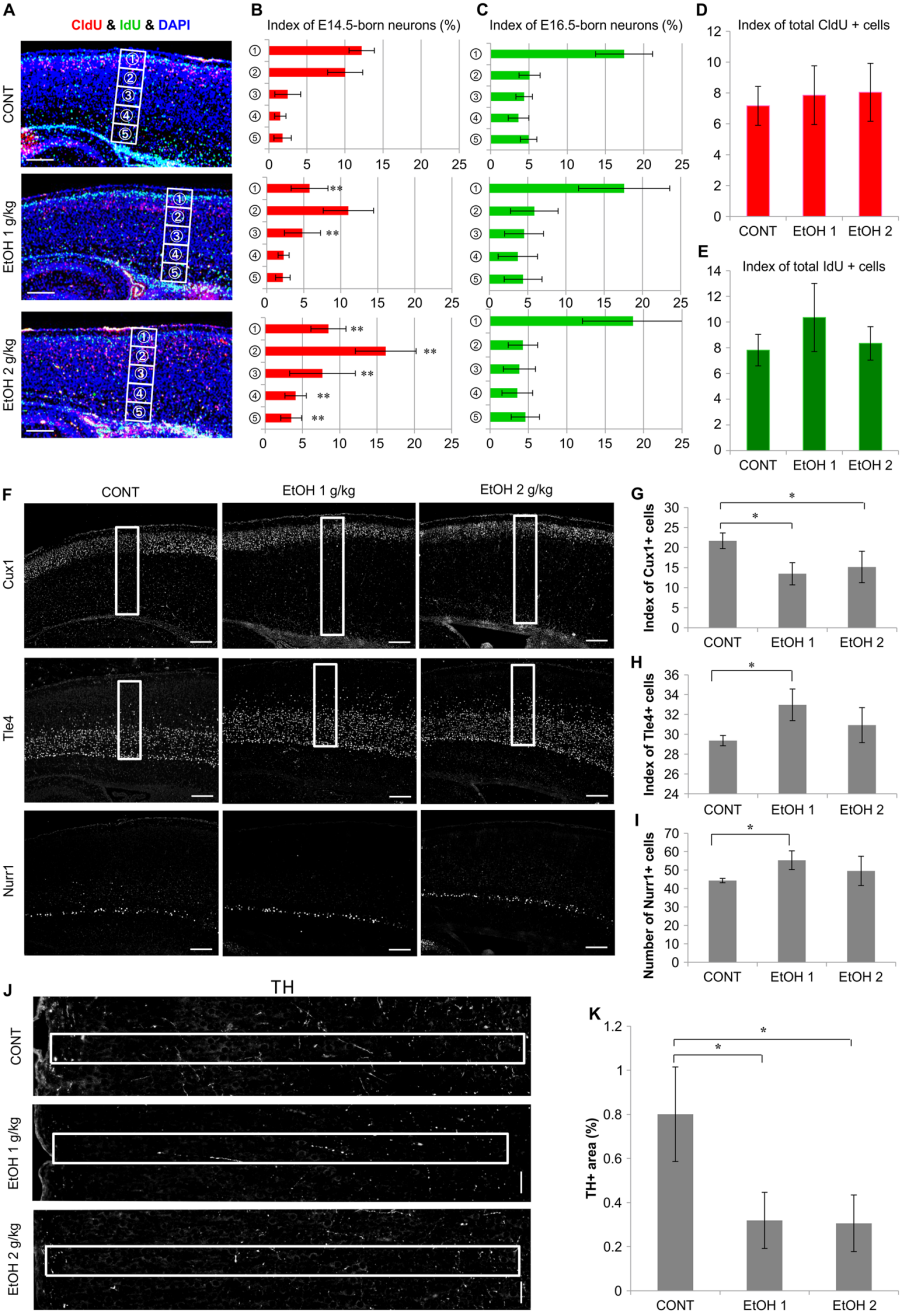
Impaired neocortical neuronal distribution, layer formation, and neuronal projection in newborn mice prenatally exposed to EtOH. (**A**) Images of birth-date analysis in parasagittal neocortical control (n = 6) and prenatal EtOH treated tissue sections (n = 6, respectively) at P3, using anti-CldU and IdU antibodies double staining after single pulse injections of CldU on E14.5 (red) and IdU on E16.5 (green). Scale bar, 100 µm. (**B**,**C**) The ratio of CldU+ (**B**) or IdU+ cells (**C**) to the total number of cells (blue DAPI + cells) in ventral to dorsal bins (white boxes in **A**) at P3. (**D**,**E**) The total number of CldU + E14.5-born (**D**) and IdU + E16.5-born neurons (**E**). (**F**) Immunostaining of P3 parasagittal neocortical sections with anti-Cux1 (layer 2/3), Tle4 (layer 4/5), and Nurr1 (layer 6b) antibodies. (**G**–**I**) The indice of neocortical Cux1+ (**G**), Tle4+ (**H**), and the number of Nurr1+ (**I**) neurons. Cells were counted in 100 µm wide sampling boxes (white boxes in **A** and **F**); scale bar, 100 µm. (n = 6, respectively). (**J**) Images of P3 parasagittal neocortical sections immunostained with anti-tyrosine hydroxylase (TH) antibody, in which boxes indicate the measured area (from layer 1–6). (n = 6, respectively). (**K**) The percentage of neocortical TH+ regions. Neurites were measured in 50 µm wide sampling boxes (white boxes in **J**); scale bar, 50 µm, *P < 0.05, **P < 0.01., compared to 0 mg∙kg^−1^ controls using Dunnett’s test or Steel’s test.

Prenatal EtOH exposure reportedly affects neuronal migration during neocorticogenesis^15^. Neuronal migration defects may thus be associated with abnormal neuronal distribution. We therefore assessed the adverse effects of prenatal EtOH exposure on the P3 neocortex using neuronal birth-date analysis using multiple thymidine analogs (CldU and IdU) (Fig. 2A; high magnification images in Sup. Fig. S2A). In newborns prenatally treated with 1 g∙kg^−1^ EtOH, the index of CldU + cells was lower in Bin 1 and in Bin 3, relative to that of controls (Fig. 2B). Newborns prenatally treated with 2 g∙kg^−1^ EtOH exhibited a higher number of CldU + cells in Bins 2–5 and a lower number of CldU + cells in Bin 1 relative to controls (Fig. 2B). Indices of E16.5-born neurons in the EtOH-treated groups were comparable to those of controls (Fig. 2C). These data suggest that prenatal EtOH exposure resulted in abnormal neuronal distribution in the neocortex of P3 newborns.

In addition, the total number of labeled neurons (CldU, E14.5 and IdU, E16.5) in each neocortical Bin (1–5) remained unaltered between the EtOH-treated and control groups (Fig. 2D,E). These results corroborate those for cell cycle exit (Fig. 1C), indicating that prenatal EtOH exposure did not affect neurogenesis in the developing neocortex at E14.5 and E16.5.

### Neocortical layer 2/3 hypoplasia in newborns prenatally exposed to EtOH

Abnormal neuronal distribution may result in improper layer formation in the newborn neocortex. To clarify whether the layered neocortical structure exhibited normal/abnormal formation in newborns prenatally exposed to EtOH, we performed immunostaining using the following layer-specific markers: anti-Cux1 (layer 2/3), anti-Tle4 (layer 4/5), and anti-Nurr1 (layer 6b) antibodies (Fig. 2F; high magnification images in Sup. Fig. S2B,C,D). The index of Cux1+ neurons in the neocortex of P3 newborns prenatally treated with EtOH (1 or 2 g∙kg^−1^) significantly decreased relative to that of controls (Fig. 2G). In mice treated with 1 g∙kg^−1^ EtOH, the index of neocortical Tle4+ and the number of Nurr1+ neurons also increased significantly relative to their respective controls. However, in mice treated with 2 g∙kg^−1^ EtOH, the index of Tle4+ and the number of Nurr1+ neurons were comparable to those of their respective controls (Fig. 2H,I). Therefore, the statistical differences between the 1 g∙kg^−1^ EtOH-treated and control groups (index of Tle4+ and number of Nurr1 + cells) had no toxicological significance due to a lack of dose-dependence. Nevertheless, these data indicate that prenatal EtOH exposure specifically affected the formation of layer 2/3 of the neocortex.

### Prenatal EtOH exposure induced reduction of dopaminergic neuronal projections into neocortex

Mesocortical dopaminergic neurons in the midbrain project to the prefrontal cortex^16^. We evaluated whether dopaminergic neuronal projections to the prefrontal cortex of newborns were affected by prenatal EtOH exposure using tyrosine hydroxylase (TH) immunostaining to mark dopaminergic neurons (Fig. 2J; high magnification images in Sup. Fig. S2E). The TH+ area in the prefrontal cortex was significantly smaller in mice treated with 1 and 2 g∙kg^−1^ EtOH than in controls (Fig. 2K). These data suggest that prenatal EtOH exposure affected the projections of TH+ neurons into the prefrontal cortex of newborns in the present study.

### Effects of prenatal EtOH exposure on microglial number, type, and morphology

**Figure 3:**
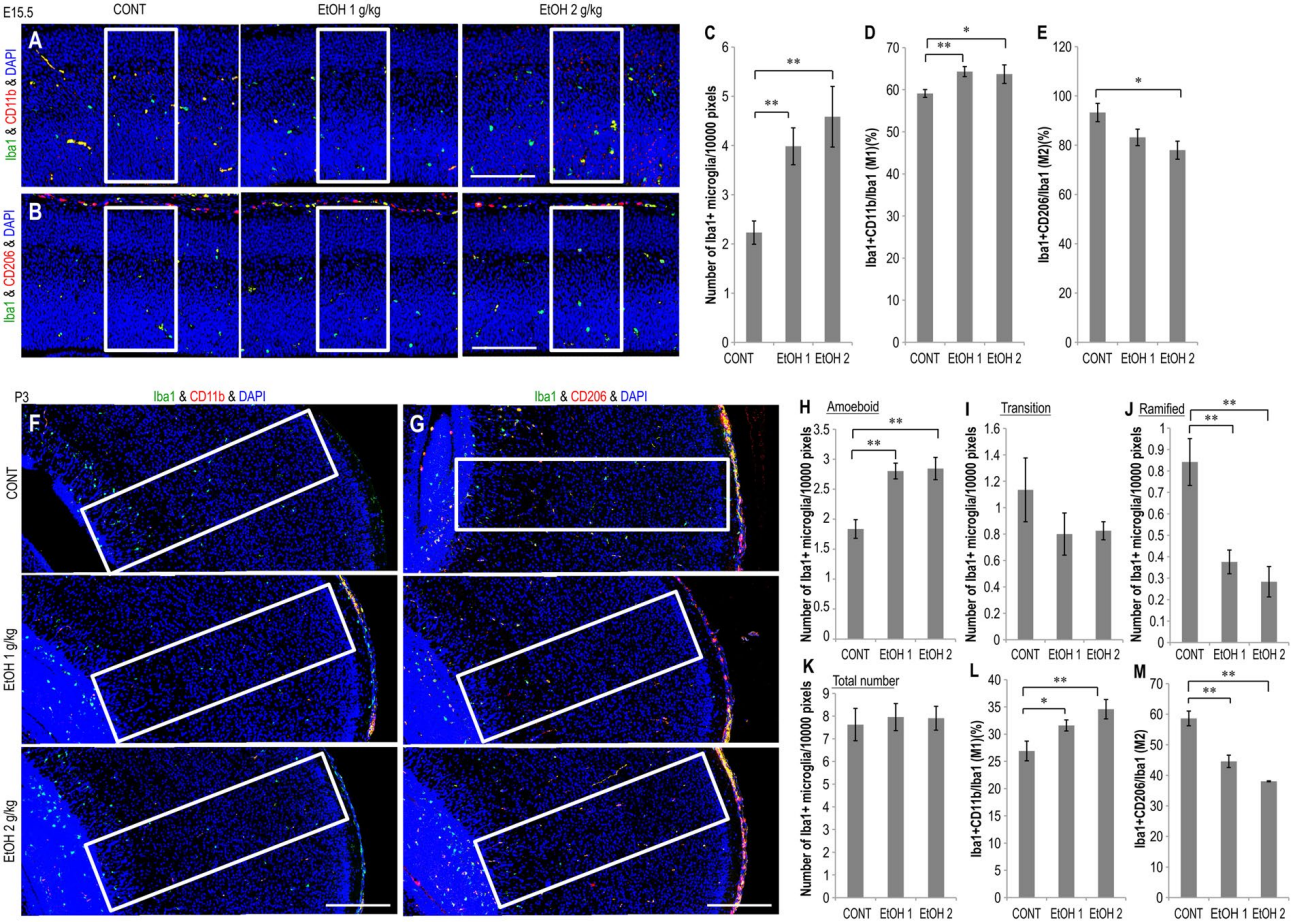
Effects of prenatal EtOH exposure on microglial number, morphology, and M1/M2 ratio in embryos and newborns. Prenatal exposure to EtOH altered microglial number and morphology and increased the ratio of M1/M2 activation in embryos and newborns. (**A**,**B**) Images of parasagittal sections at E15.5 obtained via double-immunostaining with anti-Iba1 and anti-CD11b (M1 microglial marker) or anti-CD206 (M2 microglial marker) antibodies (n = 6, respectively). (**C**) Graph depicting the index of Iba1+ microglia at E15.5. (**D**) Graph depicting the index of M1 microglia (yellow, Iba1&CD11b + cells/Iba1 + cells × 100) at E15.5. (**E**) Graph depicting the number of M2 microglia (yellow, Iba1&CD206 + cells/Iba1 + cells × 100) at E15.5. (**F**,**G**) Images depicting the Iba1+ microglial morphological classifications as observed at P3 (anti-CD11b and anti-CD206 antibodies are also shown). The morphological subtypes shown are as follows: amoeboid, active round form; ramified, resting long branching processes with a small cell body; transition, intermediate-type (see Sup. Fig. S3) (n = 6, respectively). (**H**–**K**) Graph depicting amoeboid (**D**), transition (**I**), and ramified (**J**) microglia as well as total number of Iba1+ microglia (**K**). ﻿(**L**) Graph depicting the ﻿index of M1 microglia at P3. (﻿M﻿) Graph depicting the ﻿index of M2 microglia at P3.﻿ Cells were counted in 100 µm wide sampling boxes (white boxes in **A**, **B**, **E**, and **F**); scale bar, 100 µm, *P < 0.05, **P < 0.01., compared to 0 mg∙kg^−1^ controls using Dunnett’s test or Steel’s test.

During neocortical development, microglia contribute to proper corticogenesis. Developmental stage-dependent alterations in microglial distribution and morphology have also been observed in the developing neocortex following EtOH exposure^5^. Active microglia exhibit a globular structure (amoeboid-type), while quiescent microglia have numerous processes (ramified-type), though an intermittent (transition-) type of microglia has also been observed during postnatal development^8, 17^ (Sup. Fig. S3). To determine whether abnormal microglial number, morphology, and the ratio of M1 (classically activated, inflammatory cells) to M2 microglia (reparative cells) were affected by prenatal EtOH exposure, we performed double-immunostaining of anti-Iba1 (microglial marker) using CD11b (M1 microglial marker)^18^ or CD206 (M2 microglial marker)^18^ in the neocortex of E15.5 and P3 mice (Fig. 3A,B,F,G; high magnification images in Sup. Fig. S3).

At E15.5, the number of Iba1+ amoeboid-type microglia in the dorsal telencephalon of all EtOH-treated groups increased relative to that of the respective control groups (Fig. 3C). At P3, quantification and classification of Iba1+ microglia revealed a significant increase in the number of amoeboid-type microglia in EtOH-treated mice (Fig. 3H). The number of ramified-type microglia was significantly lower in the EtOH-treated groups (Fig. 3J). However, the transition type number and total number of Iba1+ microglia were comparable between EtOH-treated groups and the control group (Fig. 3I,K).

Quantification and classification of M1 (yellow, Iba1+/CD11b+ cells, Fig. 3D,E) and M2 microglia (yellow, Iba1+/CD206+ cells, Fig. 3L,M) revealed a significant increase in the M1/M2 ratio in EtOH-treated mice at E15.5 and P3. These data suggest that prenatal EtOH exposure affected microglial number/morphology and promoted excessive M1 differentiation in the neocortex of E15.5 and P3 mice.

### Abnormal microglial profile and neuro-inflammation in the neocortex

**Figure 4:**
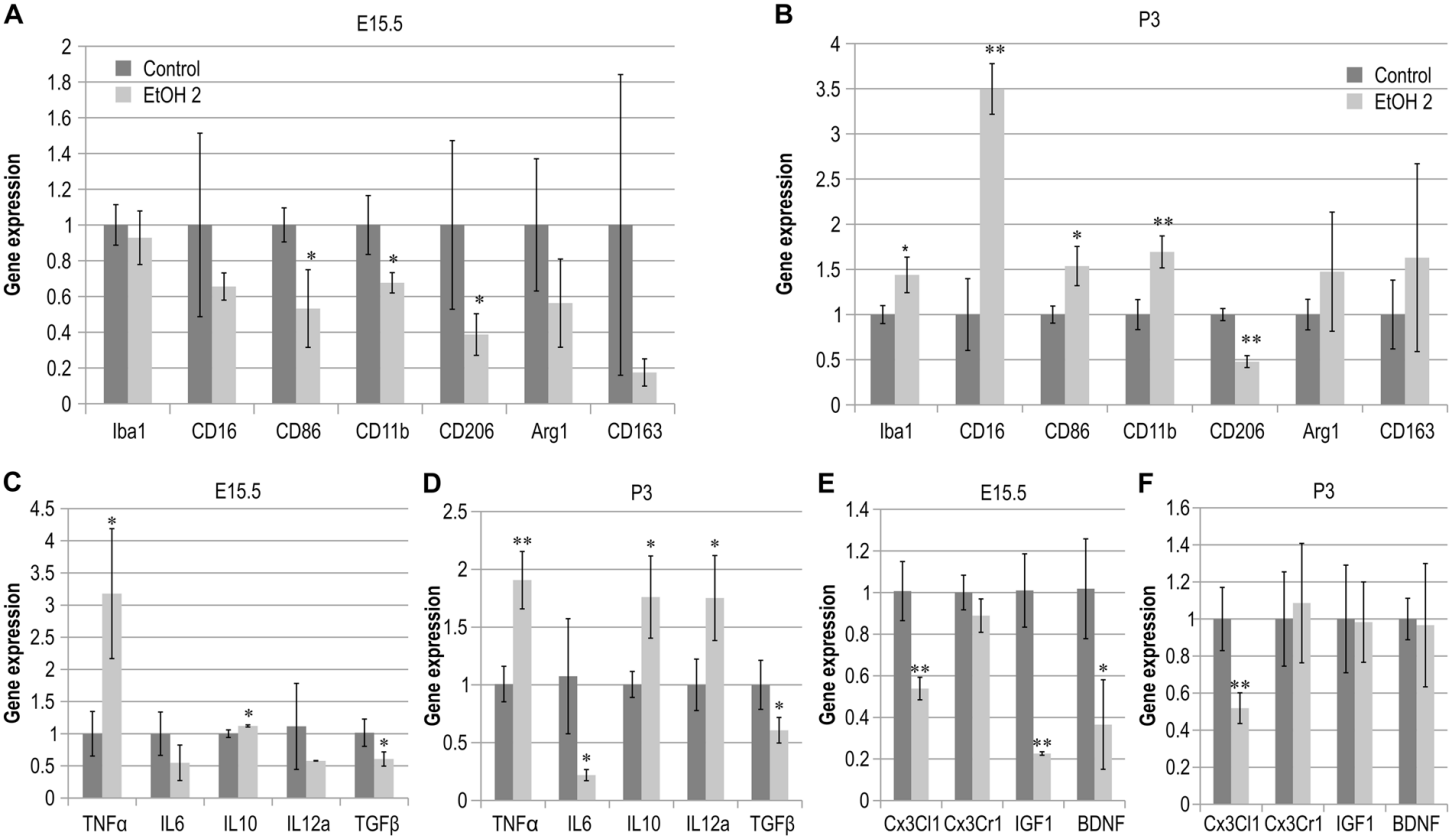
Disrupted mRNA expression of microglial markers, cytokines, and neurotrophic factors. Altered mRNA expression was observed in the embryonic dorsal telencephalon and prefrontal cortex of mice prenatally exposed to 2.0 g∙kg^−1^ EtOH (n = 6, respectively). (**A**,**B**) Graphs depicting expression of *Iba1, CD16, CD86, CD11b, CD206, Arg1*, and *CD163* mRNA in the dorsal telencephalon or prefrontal cortex at E15.5 (**A**) and P3 (**B**). (**C**,**D**) Graph depicting the expression of *TNFα, IL6*, *IL10, IL12a*, and *TGFβ* mRNA at E15.5 (**C**) and P3 (**D**) in the dorsal telencephalon and prefrontal cortex, respectively. (**E**,**F**) Graph depicting the expression of *Cx3Cl1*, *Cx3Cr1, IGF1*, and *BDNF* mRNA expression in the dorsal telencephalon and prefrontal cortex at E15.5 (**E**) and P3 (**F**), respectively. *P < 0.05, **P < 0.01 compared to 0 g∙kg^−1^ controls using Student’s t-test or Aspin-Welch’s t-test.

Because morphological anomalies of microglia in the neocortex were observed in newborn mice prenatally exposed 2 g∙kg^−1^ EtOH, we performed an analysis of microglia-related gene expression in these mice. Microglial differentiation is guided and influenced by a number of neuro-inflammatory factors, cytokines, and chemokines as well by neuron-microglia signaling. We therefore examined the differentiation of microglia by assessing expression of M1 versus M2 microglial markers in the E15.5 dorsal telencephalon and P3 prefrontal cortex (M1 markers: *CD16*, *CD86*, *CD11b*; M2 markers: *CD206*, *Arg1*, *CD163*). At E15.5, expression of *CD86, CD11b, and CD206* mRNA significantly decreased in the dorsal telencephalon following prenatal EtOH exposure (Fig. 4A). At P3, mRNA expression of microglial marker (*Iba1*) and M1 markers (*CD16, CD86* and *CD11b*) in the prefrontal cortex was significantly increased in mice prenatally exposed to 2 g∙kg^−1^ EtOH, while M2 marker expression (*CD206*) was significantly decreased (Fig. 4B). In contrast, M1 and M2 microglial marker expression decreased in the dorsal telencephalon at E15.5 in the 2 g∙kg^−1^ EtOH group.

Previous research has revealed that neuro-inflammation induced by prenatal exposure to EtOH results from cytotoxicity and the secretion of inflammatory factors^11^. Abnormal activation and differentiation of microglia may therefore be due to aberrant levels of cytokines and chemokines. Thus, we investigated whether the expression of cytokines and chemokines associated with microglial activation are influenced by prenatal 2 g∙kg^−1^ EtOH exposure. Expression of *TNFα* and *IL10* mRNA was significantly higher, while that of *TGFβ* was significantly lower, in the dorsal telencephalon at E15.5 following prenatal EtOH exposure in the 2 g∙kg^−1^ group than in controls. In contrast, no significant differences in the expression of *IL6* and *IL12a* mRNA was observed between the two groups (Fig. 4C). At P3, levels of *TNFα*, *IL10* and, *IL12a* mRNA expression were significantly increased, while levels of *IL6* and *TGFβ* mRNA expression were significantly decreased in the prefrontal cortex following EtOH exposure (Fig. 4D). These data suggest that prenatal exposure to EtOH induced abnormalities in cytokine expression in our mouse model of FASD.

### Effects of prenatal EtOH exposure on neuron-microglia signaling molecules

During the development and maturation of the neocortex, neuron and microglia interact with each other via many kinds of signal transducers (cytokines, chemokines, and neurotrophic factors)^19^. We investigated whether the expression of these signal transducers was affected by prenatal exposure to EtOH. Levels of *Cx3Cl1*, *IGF1*, and *BDNF* mRNA expression were significantly decreased relative to those of controls in the dorsal telencephalon at E15.5 following prenatal exposure to 2.0 g∙kg^−1^ EtOH (Fig. 4E). *Cx3Cr1* (receptor of fractalkine) mRNA expression was comparable between EtOH-treated and control embryos (Fig. 4E). In addition, expression of *Cx3Cl1* mRNA was significantly decreased relative to that of controls in the prefrontal cortex at P3 following prenatal exposure to 2.0 g∙kg^−1^ EtOH (Fig. 4F). *IGF1, Cx3Cr1*, and, *BDNF* mRNA expression remained unaffected following prenatal exposure to 2.0 g∙kg^−1^ EtOH (Fig. 4F). These data indicate that the expression of common neuron-microglia signal transducers and neurotrophic factors in the dorsal telencephalon and neocortex was disrupted by prenatal exposure to EtOH.

### Effect of inhibition of inflammatory chemokines/cytokines on abnormal microglial activation

**Figure 5:**
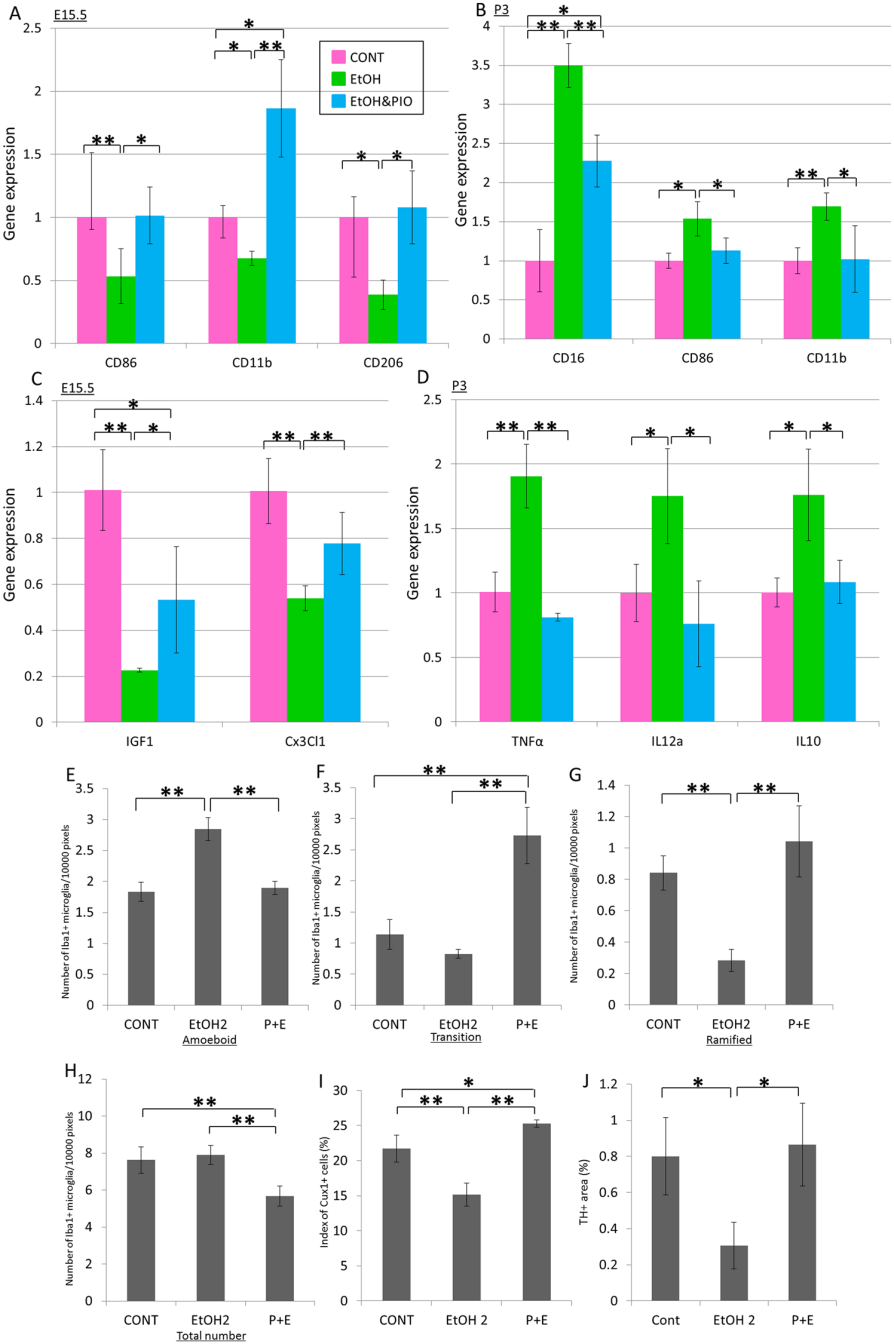
Effect of pioglitazone co-treatment on impaired mRNA expression and abnormal microglial morphology induced by prenatal exposure to EtOH. Co-treatment with pioglitazone attenuated impairments in microglia-associated mRNA expression and microglial morphology resultant from prenatal exposure to 2.0 g∙kg^−1^ EtOH (n = 6, respectively). (**A**) Graph depicting the expression of *CD86, CD11b*, and *CD206* mRNA in the dorsal telencephalon of the EtOH, E + P, and control groups at E15.5. (**B**) Graph depicting *CD16, CD86* and *CD11b* mRNA expression at P3. (**C**) Graph depicting *IGF1* and *Cx3Cl1* mRNA expression at E15.5. (**D**) Graph depicting *TNFα, IL12a*, and *IL10* mRNA expression at P3. Parasagittal sections of the prefrontal cortex were immunostained at P3 for anti-Iba1 antibody to identify and quantity microglial number and morphology. (**E**–**H**) Graphs depicting the number of amoeboid (**E**), transition (**F**), ramified (**G**), and total microglia (**H**) in the prefrontal cortex on P3. Parasagittal sections of the prefrontal cortex at P3 immunostaining for anti-Cux1 and anti-TH antibodies to identify and quantify layer 2/3 and dopaminergic neurons. Graphs depicting the indices of Cux1 + cells (**I**) and TH + area (**J**) in the prefrontal cortex at P3, *P < 0.05, **P < 0.01. One-way ANOVAs with Bonferroni’s *post hoc* test were used.

We next investigated the ability of the peroxisome proliferator-activated receptor (PPAR) γ agonist pioglitazone to block the effects of EtOH on the expression of cytokines and chemokines. PPARγ modulates inflammatory responses and controls lipid and glucose metabolism as well as energy homeostasis. PPARγ is expressed by neurons, astrocytes, oligodendrocytes, and microglia in the CNS^20,21]^. At E15.5, levels of *CD86* and *CD206* mRNA in the dorsal telencephalon of mouse embryos exposed to pioglitazone and 2.0 g∙kg^−1^ EtOH (P + E treatment) were comparable to those of controls (Fig. 5A). *CD11b* mRNA expression in P + E embryos was significantly increased relative to that of control and EtOH-treated embryos (Fig. 5A). At P3, levels of *CD16* mRNA expression in P + E mice were significantly increased relative to those of controls yet significantly decreased relative to those of EtOH-treated newborns (Fig. 5B). Levels of *CD86* and *CD11b* mRNA in P + E treated newborns were comparable to those of controls (Fig. 5B). These data suggest that pioglitazone prevented the abnormal expression of microglial markers caused by prenatal EtOH exposure.

We further observed that mRNA levels of the neuron-microglia signal transduction factors *IGF1* and *Cx3Cl1* were significantly higher in P + E treated embryos than in EtOH-treated embryos at E15.5 (Fig. 5C). Levels of *IGF1* mRNA in P + E treated embryos were significantly lower than those in the controls, though levels of *Cx3Cl1* mRNA were comparable (Fig. 5C).

Next, we assessed whether the abnormal expression of cytokines and chemokines due to prenatal EtOH exposure affects microglial activity. *TNFα*, *IL12a*, and *IL10* mRNA levels in P3 newborns prenatally exposed to P + E were significantly lower than those in EtOH-treated newborns and were comparable to those of controls (Fig. 5D). These data indicate that pioglitazone treatment prevented the abnormal expression of microglial markers associated with prenatal EtOH exposure by decreasing pro-inflammatory factors and increasing neuron-microglia signal transduction factors.

To confirm that EtOH exposure induced the observed increases in expression of pro-inflammatory molecules due to microglial activation, we examined the number and morphology of microglia in P + E newborns via immunostaining with anti-Iba1 antibody in the prefrontal cortex at P3. The numbers of amoeboid and ramified microglia in P3 newborns of the P + E group were significantly higher and lower, respectively, than those in EtOH-treated newborns. Numbers of both amoeboid and ramified microglia were comparable to those of controls (Fig. 5E and G). The number of transition microglia in the P + E group was significantly higher than that in the control and EtOH-treated groups (Fig. 5F). The total number of microglia in the P + E group was significantly lower than that in the control and EtOH-treated newborns (Fig. 5H). These data suggest that pioglitazone treatment prevented the manifestation of abnormal microglial morphology in the neocortex induced by prenatal exposure to EtOH.

We examined the expression of markers in layer 2/3 and dopaminergic neurons in P + E newborns via immunostaining with anti-Cux1 and TH antibodies, respectively, in the prefrontal cortex at P3 in order to determine whether EtOH exposure induces abnormalities in layer formation and neural projections of dopaminergic neurons. The index of Cux1 + cells in P3 newborns of the P + E group was significantly higher than that of control and EtOH-treated newborns (Fig. 5I). The index of TH + areas in the P + E group was significantly higher than that in EtOH-treated newborns and comparable to that in controls (Fig. 5J). These data suggest that pioglitazone treatment ameliorated histological abnormalities in layer formation and neuronal projections of dopaminergic neurons in the neocortex induced by prenatal exposure to EtOH.

## Discussion

In the present study, we built upon the results of previous studies by demonstrating the following effects of prenatal EtOH exposure on neocortical development: abnormal distribution of neurons across neocortical layers, layer 2/3 hypoplasia, and reductions in dopaminergic afferents. In addition, our results provide evidence that these deficits may be induced by abnormal allocation of M1 versus M2 type microglia due to aberrant expression of pro-inflammatory and neurotrophic molecules (See Sup. Fig. S4 for a model).

Defects of neocortical development due to EtOH exposure may exert widespread effects on neural function. Neocortical structures form neural networks with other brain regions to control higher cerebral functions, and EtOH cytotoxicity may directly affect neural migration and neuronal projections^15, 22^. In the present study, neuronal distribution and layer formation were disrupted, and dopaminergic neuronal projections were reduced in the neocortex of EtOH-exposed newborns at P3 (Fig. 2). In addition, our birth-date analysis revealed that EtOH exposure most significantly affected E14.5-born neurons (Fig. 2). Of particular note, some research has indicated that abnormal neocortical layer structure reduces dopaminergic neuronal projections in the prefrontal cortex of bisphenol A-treated and Smoothened conditional knockout mice^23, 24^. The mesocortical dopaminergic pathway (substantia nigra-prefrontal cortex) controls the reward system of the brain as well as behavior^16, 25, 26^. When taken with the results of these previous studies, our findings suggest that abnormalities in dopaminergic neuronal projections may be due to impaired neocortical neuronal distribution and layer formation in mice prenatally exposed to EtOH.

During neocortical maturation, microglia play an important role in the formation of neural networks, controlling neuronal migration, synaptogenesis, and neurotransmission, particularly in layer 2/3^7^. Furthermore, microglia promote neuronal survival, neurogenesis, and axonal growth by providing IGF1^27^. Squarzoni *et al*.^28^ demonstrated that microglia preferentially accumulate in specific regions of dopaminergic axonal tracts and affect axonal extension. In the present study, prenatal treatment with pioglitazone ameliorated histological abnormalities in layer 2/3 formation and dopaminergic neuronal projections associated with EtOH exposure in the neocortex at P3 (Fig. 5). In parallel, EtOH-induced activation of microglia and abnormal expression of inflammatory/microglial molecules were prevented by prenatal treatment with pioglitazone (Fig. 5). The results of the present study suggest that microglial abnormalities in P3 newborns that received prenatal treatment with EtOH may be associated with the observed defects in dopaminergic neuronal projection and neuronal distribution.

The results of the present study also indicate that prenatal exposure to EtOH excessively and specifically promotes M1 activation at the expense of M2 activation in the newborn neocortex (Fig. 3 and 4). Cx3Cl1 is expressed in neurons and is the exclusive ligand of Cx3Cr1, which is uniquely expressed in microglia of the brain. Notably, in the absence of Cx3Cr1, M1 microglial activation is significantly higher in mutant SOD transgenic mice^29^. Cx3Cl1-Cx3Cr1 signaling induces the activation of M2 microglia and promotes the release of neurotrophic factors (IGF1 and BDNF) for neuroprotection^10^. These data suggest that Cx3Cl1 is a candidate effector of abnormal microglial differentiation and activation associated with prenatal EtOH induced cytotoxicity.

PPARγ is a member of the nuclear receptor family for steroids/thyroid hormones and is expressed in the neurons, astrocytes, oligodendrocytes, and microglia of the CNS^20^. PPARγ agonists inhibit activation of microglia and production of pro-inflammatory factors^30–32^. We determined that inhibition of PPARγ with pioglitazone attenuated abnormalities in microglial development and M1/M2 ratio in mice prenatally exposed to EtOH, in accordance with the results of previous studies^11, 13^. This result also coincided with the reversal of aberrant mRNA expression of pro-inflammatory cytokines (*TNFα* and *IL12a*) and microglial markers (*CD16, CD86, CD11b*, and *CD206*). When taken together, these data indicate that the neuroprotective and anti-inflammatory effects of pioglitazone may ameliorate abnormal microglial activation and brain inflammation associated with prenatal exposure to EtOH, presenting a novel target for the prevention of FASD.

Abnormal microglial distribution, function, and activation have been documented in various developmental and mental disorders, including autism, schizophrenia, and Rett syndrome^7, 33–36^. Defective microglial function has also been associated with reductions in social behavior^37^. Patients with FASD have higher rates of mental disorders, mental retardation, hyperactivity, sensitivity to stress, and ADHD, which are likely the result of morphological defects in the brain and in the formation of neural networks. Our mouse models of FASD exhibited similar anatomical and cellular deficits. We therefore hypothesize that these anomalies underlie symptoms of FASD and interact with each other during neocortical development in mouse models of FASD. We thus propose two hypothetical bases for prenatal EtOH exposure-induced abnormal behaviors. First, aberrations in neuron distribution and layer formation result in altered patterns of dopaminergic neuronal projection in newborns prenatally exposed to EtOH, thereby producing abnormalities in behavior. Second, increased M1 activation along with decreased M2 activation may affect neocortical synaptogenesis and neurotransmission.

In the prenatal stage (Sup. Fig. S4A), EtOH exposure induced excessive cell death, increased the number of microglia, and produced brain inflammation in the dorsal telencephalon. In the postnatal stage (Sup. Fig. S4B), the proliferation of neural stem cells and expression of neurotrophic factors (BDNF and IGF1) were also decreased in newborns that had been prenatally exposed to EtOH, inducing disruptions in cytokine expression. Furthermore, neuronal expression of Cx3Cl1 decreased following prenatal exposure to EtOH, contributing to the abnormal pattern of increased M1 activation and decreased M2 activation. These results suggest that neuronal degradation and abnormal microglial differentiation result in morphological abnormalities in the neocortex of newborns.

Various environmental or industrial chemicals that affect the developing brain, including EtOH, have the potential to induce neurodevelopmental or mental disorders. The neurodevelopmental toxicity of these chemicals may be associated with increased neuro-inflammation during brain development. In the present and previous studies^11^, anti-inflammatory drugs have been observed to ameliorate abnormal levels of inflammation, microglial conditions, and histological anomalies in the neocortex following maternal exposure to EtOH during pregnancy. The results of the present study suggest that anti-inflammatory agents may be effective in the treatment of neurodevelopmental disabilities (e.g., FASD) resulting from prenatal exposure to industrial chemicals.

Previous studies have revealed that prenatal EtOH exposure affects the cytoarchitecture of the rodent neocortex during development, and that pioglitazone blocks EtOH-induced microglial activation and neuro-inflammation^11^. The present study provides the first evidence that prenatal EtOH exposure affects microglial differentiation and proliferation by inducing abnormalities in the expression of neuron-microglia signaling molecules. In addition, our findings suggest that the cytoarchitecture of the neocortex can be restored via treatment with anti-inflammatory agents that target microglial abnormalities and neuro-inflammation. These findings suggest that abnormal behaviors generally observed in FASD may result from morphological and functional abnormalities of the neocortex, perhaps due to neuro-inflammation, dysregulation of microglial development, and improper morphogenesis of the neocortex. Further studies are required in order to address the correlation between these findings and behavioral phenotypes.

## Methods

### Prenatal EtOH treatment regimen

All procedures in the present study conformed to the principles outlined in the Guide for the Care and Use of Laboratory Animals published by the US National Institutes of Health (NIH Publication No. 85–23, revised 1996 and updated 2011) and were approved by the Institutional Animal Care and Use Committee of Kindai University (approval number: KASE-26–003). Twenty-five 9-week-old male ICR mice and 60 8-week-old female ICR mice were purchased from CLEA (Osaka, Japan) for use in the experiments of the present study. EtOH was purchased from Wako Pure Chemical Industries (Tokyo, Japan) and diluted with distilled water (Otsuka Pharmaceuticals, Tokyo, Japan). The *in vivo* FASD animal model was established according to a method previously described by Deng and Elberger^38^. Briefly, pregnant females were administered EtOH (25% w/v) via intragastric gavage at a dose of 1 or 2 g∙kg^−1^ body weight twice daily (12:00 pm and 6:00 pm) from E6 through E18. Food and water were removed from all dams, including controls, at 10:00 am and 4:00 pm to clear the stomach of chyme and facilitate absorption of EtOH. Food and water were replaced approximately 2 h following intubation. The solutions were prepared prior to each dosing and administered at defined times (12:00–12:15 pm and 6:00–6:15 pm). Control animals received similar volumes of fluid via an isocaloric substitution of sucrose for EtOH at 2 g∙kg^−1^. There were no significant differences in body or brain weight between control mice (paired group) and those exposed to either 1 or 2 g∙kg^−1^ EtOH in a previous study^39^. We did not include a chow-fed group in the present study because no meaningful differences in corticogenesis between pair-fed and chow-fed groups have been observed in previous studies^3, 13^. Downing *et al*.^40^. reported that the blood ethanol levels of pregnant mice intubated twice daily with 3.0 g∙kg^−1^ EtOH from E7 to E18 averaged 317 mg∙dl^−1^ 30 min after the second administration and decreased to 56–117 mg∙dl^−1^ at 180 min.

### Immunofluorescence and cell counting

For analysis, we used either six embryos or newborns from three dams in each group or positively stained areas in six anatomically matched sections from two embryos and two newborns from different dams. Cells were quantified using Adobe Photoshop CS4 software (Adobe, San Jose, CA, USA), as previously described^24^. In the dorsal telencephalon/neocortex, cells positive for ionized calcium-binding adapter molecule 1 (Iba1), 5-chloro-2′-deoxyuridine (CldU), 5-iodo-2′-deoxyuridine (IdU), antigen KI-67 (Ki67), nuclear receptor related 1 (Nurr1), cut-like homeobox 1 (Cux1), transducin-like enhancer of split 4 (Tle4), CD206, CD11b, active & pro caspase-3, and 4′,6-diamidino-2-phenylindole (DAPI) were manually counted. Tyrosine hydroxylase (TH) expression area was measured in the white box (Fig. 2J) using Adobe Photoshop CS4.

These measurements were performed in 50 or 100 µm wide frames covering a region from the ventricle to the pial surface of the dorsal telencephalon/neocortex in each section. In Fig. 1A only, 100 µm wide frames (white box in Fig. 1A) covered a region from the ventricle to the proliferative zone of dorsal telencephalon (ventricular and subventricular zone). Measurement frames are depicted in Fig. 1A, 2A, 2F, 2J, 3A, 3B, 3F, and 3G. The numbers of Iba1+ microglia, active & pro-caspase-3+, and Nurr1+ cells were counted in anatomically matched regions from the anterior to posterior dorsal telencephalon at E15.5 or P3 (Fig. 1 and 2). The numbers of positive cells (number of marker-positive cells per number of DAPI + cells × 100) and positive areas (TH expression area per frame area × 100) were calculated as ratios. The number of positive cells in each section (active & pro-caspase-3 and Nurr1: number of marker-positive cells in section per l0,000 pixels of dorsal telencephalon) was also calculated.

### CldU and IdU incorporation, cell cycle exit, and birth-date analysis

Cell cycle kinetics analysis (cell cycle exit) and birth-date analysis using thymidine analogs (CldU and IdU) were performed according to previous reports^23, 24, 41, 42^. Detailed methods are described in the supplemental materials.

### RNA extraction and quantitative PCR

RNA was extracted from the anterior dorsal telencephalon on E15.5 and prefrontal cortex on P3, or freshly isolated using ReliaPrep RNA Tissue Miniprep System (Promega, Madison, WI, USA). Total RNA concentration was determined using a Nano drop (Thermo scientific, Waltham, MA, USA). Complementary DNA was synthesized from 1 µg total RNA using the QuantiTect Reverse Transcription kit (Qiagen, Hilden, Germany), in accordance with manufacturer instructions. Quantitative PCR was performed using an ABI StepOne Real Time PCR System (ABI, Foster city, CA, USA) and SYBR Fast qPCR Mix (Takara Bio. Shiga, Japan). The primer sequences are provided in Supplemental Table 2 and were designed to span introns whenever possible, and a number of these sequences have also been used in previous studies (*TNFα* and *IL6* ^43^, *GAPDH* ^44^). Ct values from triplicate measurements of each sample were averaged, and relative expression levels were determined using the ΔΔCt method. The expression of each gene was normalized to the levels of *GAPDH* within each sample.

### Treatment with pioglitazone, an anti-inflammatory agent

To determine whether prenatal EtOH exposure induces neuro-inflammation and results in abnormal microglial activation, we examined the effect of the anti-inflammatory agent pioglitazone on EtOH exposure. Pregnant females were administered either 12.5 mg∙kg^−1^ pioglitazone (5-[[4-[2-(5-ethyl-2-pyridinyl)ethoxyl]phenyl]methyl-2,4-thiazolidinedione], Cas No., 111025-46-8, Cayman Chemical, Ann Arbor, MI, USA) or water by gavage on E6 through E18, and were allowed to give birth naturally. Pioglitazone or distilled water was administered 2 hours prior to daily treatment (12:00 pm) with EtOH. The treatment regimen and dose of pioglitazone were determined according to the methods reported by Drew *et al*.^11^. A group of pioglitazone and vehicle mice was not included in this study because previous studies^11, 13^ and our preliminary analysis revealed a lack of effect from pioglitazone alone in a similar model of FASD. Offspring from four to five pregnant females in each group were allocated to the EtOH group, pioglitazone plus EtOH group, pioglitazone group, or distilled water vehicle group for histology and qPCR analysis.

### Statistical analysis

Data for body weight, brain weight, immunohistochemical analysis, and qPCR were analyzed using the litter as the experimental unit. Bartlett’s test for equal variance was used to determine whether the variance was homogenous between the control and EtOH-exposed groups with regard to the following data: numerical data (e.g., body weights and brain weights), numbers of immunoreactive cells, and levels of gene expression^45^. If the variance was homogenous, Dunnett’s test was used to compare numerical data between each EtOH group and the control group. Steel’s test^46^ was used for heterogeneous data.

In the case of data consisting of two sample groups, numerical data were assessed using the F-test for homogeneity of variance, and Student’s *t*-test was applied when the variance was homogenous between the groups using a test for equal variance^43^. If a significant difference in variance was observed, Aspin-Welch’s *t*-test was then performed. In the pioglitazone co-administration experiment, one-way analysis of variance (ANOVA) with Bonferroni’s *post hoc* test was used to analyze data for the number of immunoreactive cells as well as that for levels of gene expression^44^. A probability level of p < 0.05 was considered statistically significant in all analyses.

## Electronic supplementary material

Supplementary Materials
