# Supplementary material for: Mechanisms underlying neuro-inflammation and neurodevelopmental toxicity in the mouse neocortex following prenatal exposure to ethanol
Source: Sci Rep. 2017 Jul 10;7:4934. doi: 10.1038/s41598-017-04289-1 (PMC5504035; doi:10.1038/s41598-017-04289-1)
Supplement: Supplementary file 1 — Supplementary Materials [file 41598_2017_4289_MOESM1_ESM.pdf]

Mechanisms underlying neuroinflammation and neurodevelopmental toxicity in the mouse neocortex following prenatal exposure to ethanol

Munekazu Komada, Nao Hara, Satoko Kawachi, Kota Kawachi, Nao Kagawa, Tetsuji Nagao, and Yayoi Ikeda

Supplementary Material

Supplemental Table 1. Number of mice used in each experiment.

Group	Strain	Body weight	Brain weight	Histological analysis / qPCR	
Age		P3	P3	E15.5	P3
Control	ICR	♂: 18/6 dams	♂: 18/6 dams	♂: 6/3 dams	♂: 6/3 dams
EtOH 1.0 g/kg/day	ICR	♂: 18/6 dams	♂: 18/6 dams	♂: 6/3 dams	♂: 6/3 dams
EtOH 2.0 g/kg/day	ICR	♂: 18/6 dams	♂: 18/6 dams	♂: 6/3 dams	♂: 6/3 dams

The number of mice (embryos, newborns, and adult).

Supplemental Table 2. Primer sequences used for qPCR

Gene		Sequences	Reference
Iba1	Forward	GGACAGACTGCCAGCCTAAG	
	Reverse	GACGGCAGATCCTCATCATT	
CD16	Forward	TGTTTGCTTTTGCAGACAGG	
	Reverse	TGCTCCATTTGACACCGATA	
CD86	Forward	CACGAGCTTTGACAGGAACA	
	Reverse	TTAGGTTTCGGGTGACCTTG	
CD11b	Forward	GACTCAGTGAGCCCCATCAT	
	Reverse	AGATCGTCTTGGCAGATGCT	

CD206	Forward	ATGCCAAGTGGGAAAATCTG	
	Reverse	TGTAGCAGTGGCCTGCATAG	
Arg1	Forward	GTGAAGAACCCACGGTCTGT	
	Reverse	CTGGTTGTCAGGGGAGTGTT	
CD163	Forward	CATGTGGGTAGATCGTGTGC	
	Reverse	TGTATGCCCTTCCTGGAGTC	
TNFα	Forward	TGTAGCCCACGTCGTAGCAA	Crain et al. 2013[5]
	Reverse	AGGTACAACCCATCGGCTGG	
IL6	Forward	ACTTCCATCCAGTTGCCTTC	Crain et al. 2013[5]
	Reverse	GTCTCCTCTCCGGACTTGTG	
IL10	Forward	CCAAGCCTTATCGGAAATGA	
	Reverse	TTTTCACAGGGGAGAAATCG	
IL12α	Forward	CATCGATGAGCTGATGCAGT	
	Reverse	CAGATAGCCCATCACCCTGT	
TGFβ1	Forward	TTGCTTCAGCTCCACAGAGA	
	Reverse	TGGTTGTAGAGGGCAAGGAC	
Cx3Cl1	Forward	CGCGTTCTTCCATTTGTGTA	
	Reverse	CTGTGTCGTCTCCAGGACAA	
Cx3Cr1	Forward	TGAGTGACTGGCACTTCCTG	
	Reverse	GGACAGGAAGATGGTTCCAA	
IGF1	Forward	TGGATGCTCTTCAGTTCGTG	
	Reverse	GTGGGGCACAGTACATCTCC	
BDNF	Forward	TTGTTTTGTGCCGTTTACCA	
	Reverse	GGTAAGAGAGCCAGCCACTG	
GAPDH	Forward	TCACCACCATGGAGAAGGC	Aich et al. 2012[6]
	Reverse	GCTAAGCAGTTGGTGGTGCA	

Supplemental Methods

Animals and housing

Twenty-five 9-week-old male ICR mice and 60 8-week-old female ICR mice were purchased from CLEA (Osaka, Japan) for use in the experiments of the present study, following a 2-week acclimation period. Mice were kept under specific pathogen-free (SPF) conditions and housed in polycarbonate cages in an environment-controlled room at a temperature of 23 ± 1 °C, humidity of 50 ± 5%, and under a 12:12 h light-dark cycle (lights on at 7 a.m.). Mice were allowed free access to food (Certified Rodent Chow CE-2; CLEA, Osaka, Japan) and drinking water. Certification analysis of each lot of the diet was performed by the manufacturer. Ten- or 11-week-old virgin female mice were cohabited for 2 h (7:00–9:00 am) on a 1:1 basis with males aged 11 weeks or older. Females were checked for the presence of a vaginal plug immediately after. The presence of a plug represented embryonic day (E) 0. Twenty pregnant mice in each group were allowed to give birth and nurse their pups until postnatal day (P) 3. The day of birth was designated as P0. On the morning of P1, the number of pups in a litter was adjusted to 4–5 males and females each (total number after adjustment was 8). Pups were weighed on P1 and P3, and the number of pups in each litter during the lactation period was recorded, following which the viability on P3 after adjustment was determined. The number of mice in each experiment is detailed in Supplemental Table 1.

Tissue preparation

For histologic evaluation of embryos, pregnant mice were euthanized by cervical dislocation and subjected to cesarean section on E15.5. For histologic evaluation of newborns, mice were humanely euthanized by exsanguination under anesthesia with sevoflurane (gas concentration: 2.5–4%, Maruishi Pharmaceutical Co., Ltd., Osaka, Japan) on P1 or P3. Subsequently, the tissue samples were dehydrated using 70%, 95%, and 100% ethanol (EtOH) and xylene and then embedded in paraffin. Certain parasagittal paraffin sections (6

μm) were immunostained using antibodies for several markers. The dorsal telencephalons (neocortex) of embryos on E15.5 and newborns on P1 and P3 were histologically examined. Three E15.5 embryos and P3 pups from two dams in each group were used (Supplementary Table 1). Anatomically matched sagittal sections 0.5 mm and 2.0 mm from the craniofacial midline were used for E15.5 embryos and P3 newborns, respectively. Immunofluorescence staining of the sections was performed as previously described[1]. Tissue sections were incubated with primary and secondary antibodies overnight and for 3 h at room temperature, respectively. Nuclei were then counterstained with DAPI (D9542, Sigma-Aldrich, St. Louis, MO, USA; 1:1000) for 3 h at room temperature.

Antibodies

The following primary antibodies were used: anti-Iba1 (1:500, 019-19741, Wako, Osaka, Japan), anti-Ki67 (1:200, RM9106, Thermo Fisher Scientific, Waltham, MA, USA), anti-CldU (1:50, chlorodeoxyuridine)/BrdU (bromodeoxyuridine, BU1/75, AbD Serotec, Kidlington, UK), anti-IdU (iododeoxyuridine)/BrdU (1:50, clone B44, 347580, BD Biosciences, San Jose, CA, USA), anti-Cux1 (1:200, sc-13024, Santa Cruz Biotechnology, Dallas, TX, USA), anti-Nurr1 (1:200, AF2156, R&D Systems, Minneapolis, MN, USA), anti-Tle4 (1:200, sc13377, Santa Cruz Biotechnology), anti-tyrosine hydroxylase (TH, 1:500, 657012, Millipore, Darmstadt, Germany), anti-active + pro caspase-3 (1:200, ab13585, Abcam, Cambridge, UK). The following secondary antibodies were used: Alexa 488 and 568 anti-rabbit, mouse, and rat IgG (A11034, A11011, A11001, A11004, A11006, A11077, Thermo Fisher Scientific).

CldU and IdU incorporation, cell cycle exit, and birth-date analysis

For *in vivo* labeling of cells in the S-phase and those exiting the cell cycle, IdU (I7125, Sigma-Aldrich, St. Louis, MO, USA, 50 $mg \cdot kg^{-1}$ body weight) was intraperitoneally injected into pregnant mice 24 h prior to sampling the embryos on E15.5. The percentage of cells

exiting the cell cycle in the dorsal telencephalon was estimated from the ratio of IdU+/Ki67- (post-mitotic) cells to all IdU+ cells labeled by a 24-h pulse of IdU[2]. For neuronal birth-date analysis, pregnant females in each group were injected intraperitoneally with CldU at 50 mg·kg^{-1} body weight (MP Biomedicals, Salon, OH, USA) on E14.5 and with IdU at 50 mg·kg^{-1} body weight on E16.5 to label the neurons generated at each stage (CldU-E14.5 and IdU-E16.5). CldU and IdU were administered at 12:00 pm. On P3, brains from newborns were sampled. Rat monoclonal anti-CldU and mouse monoclonal anti-IdU antibodies were used. Immature neurons were quantified, and their distribution in the neocortex was analyzed in anatomically matched sections from each newborn according to previously reported methods[1,3,4]. The entire cortical thickness of parasagittal sections was subdivided equally into five 100-µm wide fractions (Bins 1–5, Fig. 2). CldU+ and IdU+ cells were counted, and the index (number of CldU+ or IdU+ cells/number of DAPI+ cells × 100) was calculated.

Supplemental Figures

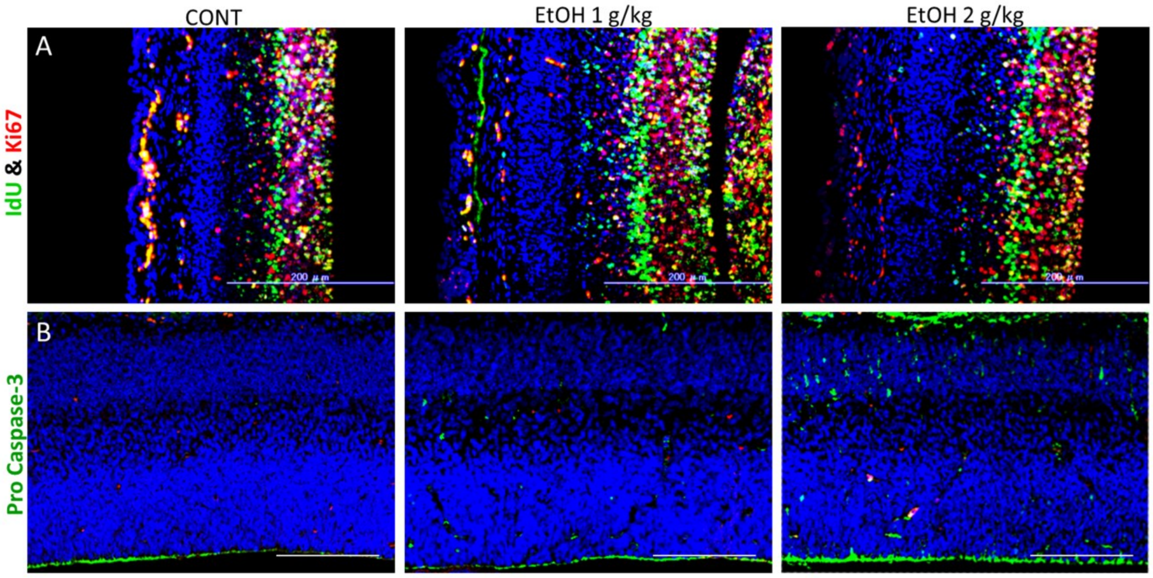

Supplemental Figure S1. High magnification images of Figure 1.

A and B are a high magnification of Figure 1A and D, respectively. Scale bar, 100 μm

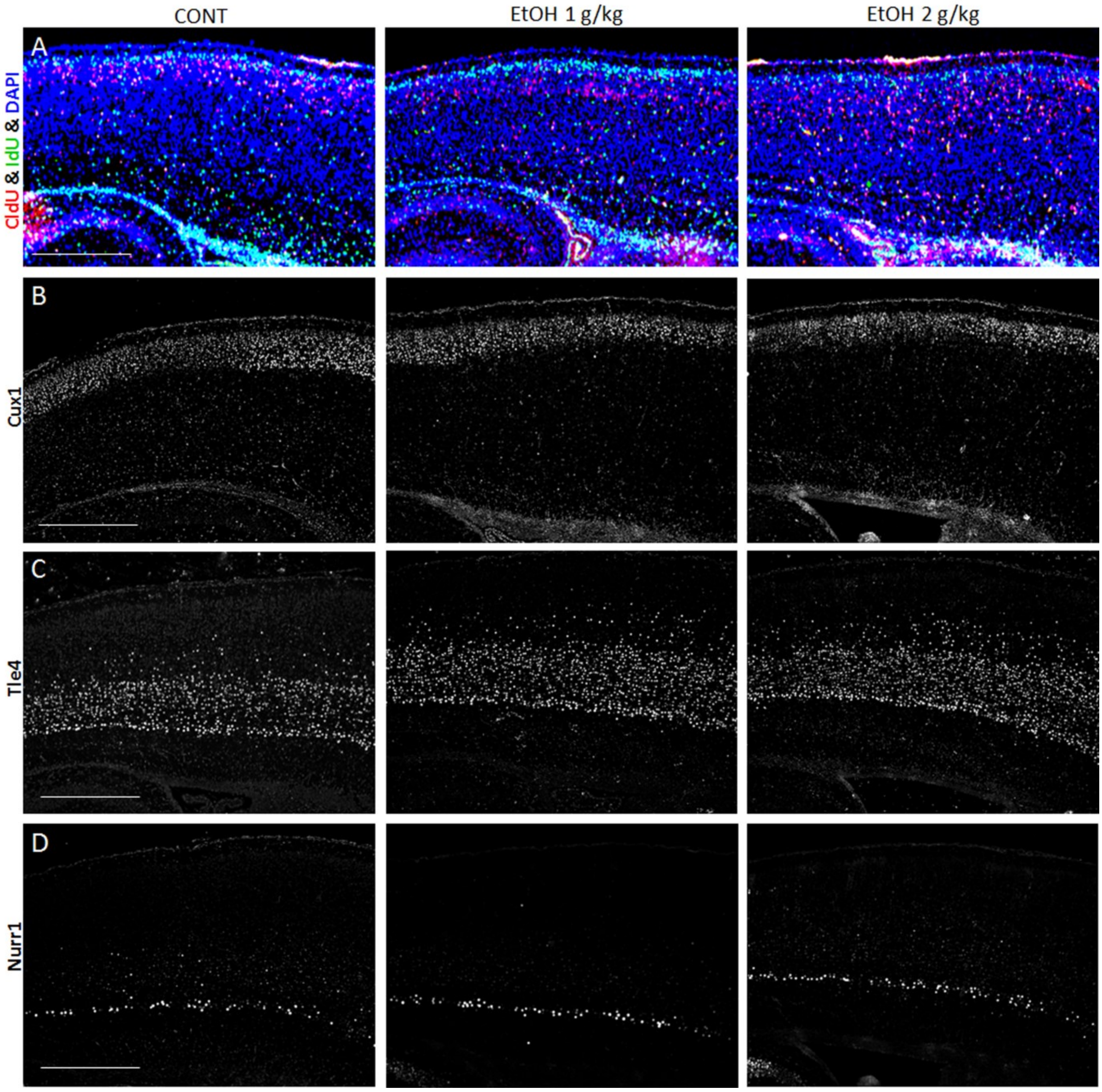
CONT
EtOH 1 g/kg
EtOH 2 g/kg
A
CldU & IdU & DAPI
B
Cux1
C
Tle4
D
Nurr1

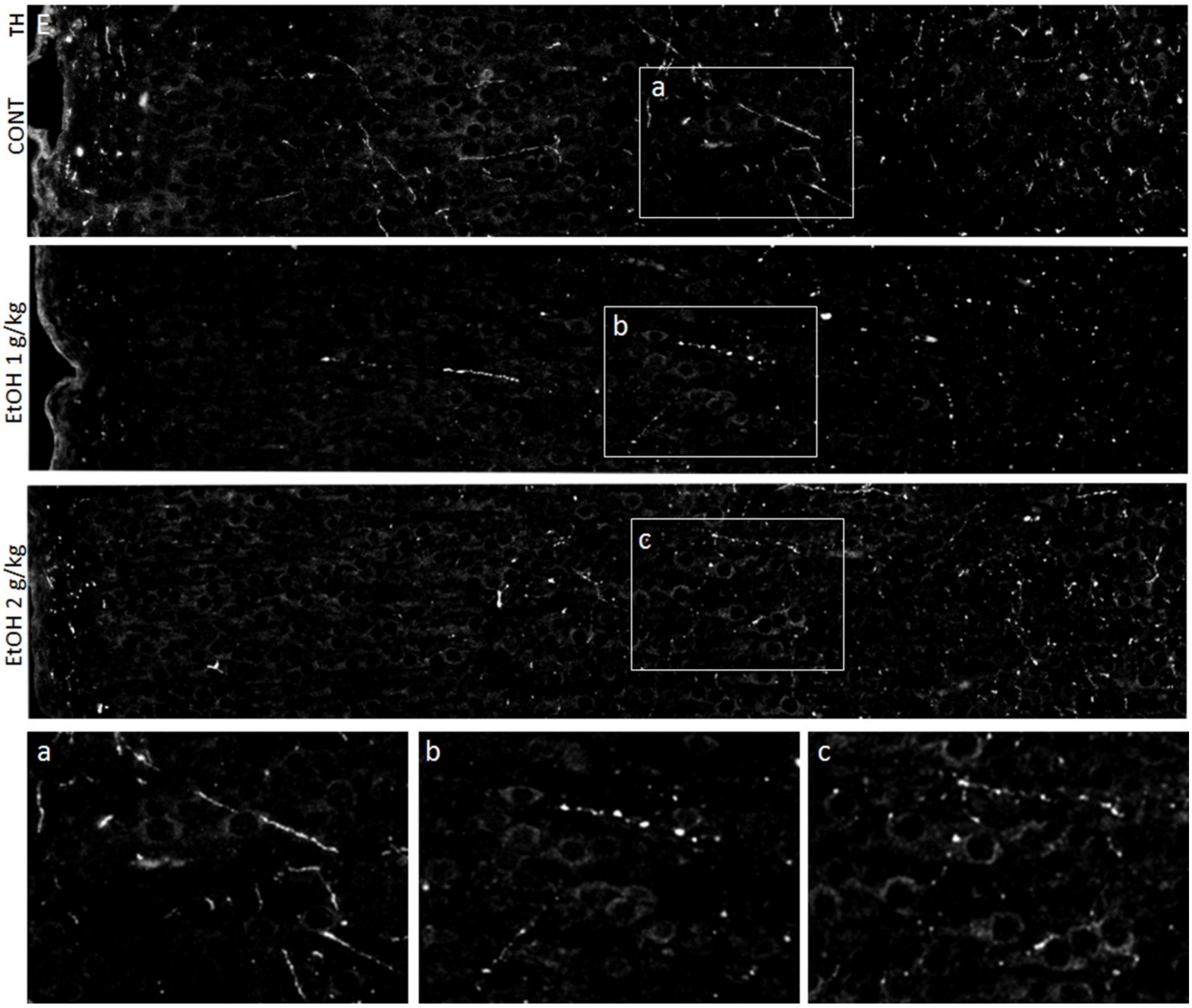

Supplemental Figure S2. High magnification images of Figure 2.

A, B, C, D, and E are high magnification images of Figure 2A, F, and J, respectively. (E) White boxes (a–c) indicate the location of high magnification images presented at the bottom. Scale bar, 100 μm

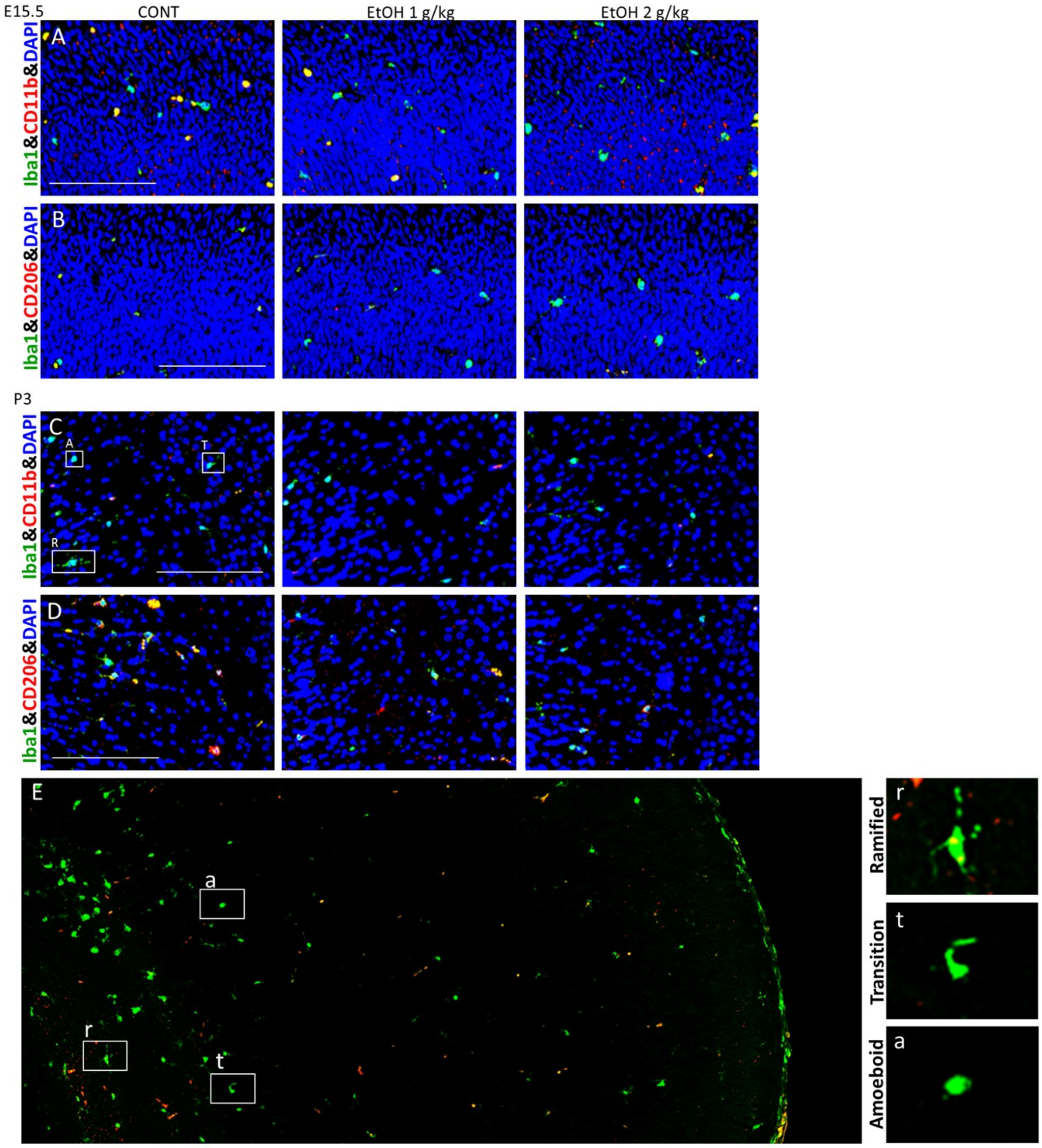

Supplemental Figure S3. High magnification images of Figure 3.

A, B, C, and D are high magnification images of Figure 3A, B, F, and G, respectively. The images show the Iba1+ microglia. (E) The image is a high magnification of Figure 3F (EtOH 2 g/kg group, Iba1 & CD11b staining). Active microglia show a globular structure (amoeboid-type, white box, a), intermission microglia have numerous processes and small

cell body (ramified-type, white box, r), and intermediate glia with one or two processes are called transition-type (white box, t). Scale bar, 100 μm

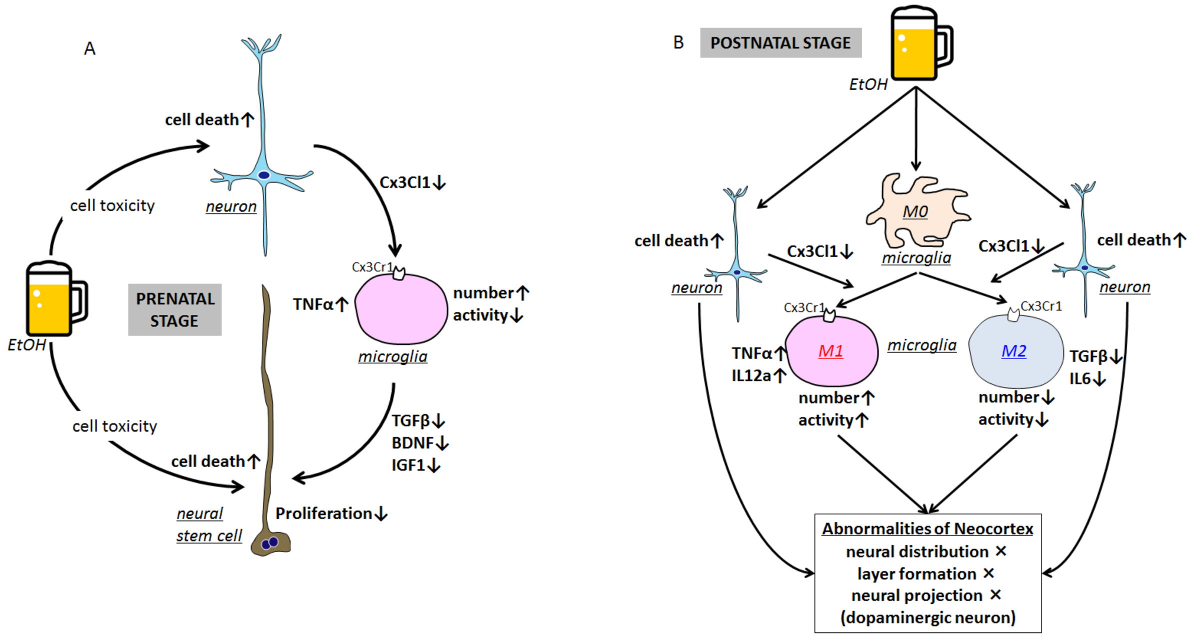

Supplemental Figure S4. Model of the pathological mechanism of FASD in the present study.

(A) In prenatal stages, cytotoxic EtOH exposure induces cell death of neurons and neural stem cells. *Cx3Cl1*, which controls microglial differentiation and activation, is expressed in cortical neurons. Levels of mRNA expression are reduced in the prenatal EtOH-treated embryos. Down-regulation of *Cx3Cl1* reduces the activation of and increases the number of microglia. Reduced microglial activation inhibits the expression of neurotrophic factors (TGFβ, BDNF, and IGF1). Thus, the proliferation of neural stem cells decreases in the dorsal telencephalon of EtOH-treated embryos. (B) At postnatal stages, prenatal EtOH exposure induces microglial activation by similar mechanisms. Down-regulation of *Cx3Cl1* induces the activation of M1 microglia and inhibition of M2 microglia. This disruption of microglial activation increases the expression of TNFα and IL12a (M1 microglial inflammation factors)

and decreases that of TGFβ and IL6 (M2 microglial inflammation factors). Abnormal microglial activation and the resultant effects on neural stem cell survival and proliferation produce morphological abnormalities in the neocortex of newborns.

Supplemental References

1 Komada, M. *et al.* Hedgehog signaling is involved in development of the neocortex. *Development* **135,** 2717-2727 (2008).

2 Chenn, A. & Walsh, C. A. Regulation of cerebral cortical size by control of cell cycle exit in neural precursors. *Science* **297,** 365-369 (2002).

3 Komada, M., Iguchi, T., Takeda, T., Ishibashi, M. & Sato, M. Smoothened controls cyclin D2 expression and regulates the generation of intermediate progenitors in the developing cortex. *Neurosci. Lett.* **547,** 87-91 (2013).

4 Komada, M. *et al.* Newborn mice exposed prenatally to bisphenol A show hyperactivity and defective neocortical development. *Toxicology* **323,** 51-60 (2014).

5 Crain, J. M., Nikodemova, M. & Watters, J. J. Microglia express distinct M1 and M2 phenotypic markers in the postnatal and adult central nervous system in male and female mice. *J. Neurosci. Res.* **91,** 1143-1151 (2013).

6 Aich, J., Mabalirajan, U., Ahmad, T., Agrawal, A. & Ghosh, B. Loss-of-function of inositol polyphosphate-4-phosphatase reversibly increases the severity of allergic airway inflammation. *Nat. Commun.* **3,** 877 (2012).
